# Construction and Analysis of High-Ethanol-Producing Fusants with Co-Fermentation Ability through Protoplast Fusion and Double Labeling Technology

**DOI:** 10.1371/journal.pone.0108311

**Published:** 2014-09-30

**Authors:** Jingping Ge, Jingwen Zhao, Luyan Zhang, Mengyun Zhang, Wenxiang Ping

**Affiliations:** Key Laboratory of Microbiology, College of Life Science, Heilongjiang University, Harbin, P. R. China; University of Groningen, The Netherlands

## Abstract

Double labeling of resistance markers and report genes can be used to breed engineered *Saccharomyces cerevisiae* strains that can assimilate xylose and glucose as a mixed carbon source for ethanol fermentation and increased ethanol production. In this study *Saccharomyces cerevisiae* W5 and *Candida shehatae* 20335 were used as parent strains to conduct protoplast fusion and the resulting fusants were screened by double labeling. High performance liquid chromatography (HPLC) was used to assess the ethanol yield following the fermentation of xylose and glucose, as both single and mixed carbon sources, by the fusants. Interestingly, one fusant (ZLYRHZ7) was demonstrated to have an excellent fermentation performance, with an ethanol yield using the mixed carbon source of 0.424 g g^−1^, which compares with 0.240 g g^−1^ (W5) and 0.353 g g^−1^ (20335) for the parent strains. This indicates an improvement in the ethanol yield of 43.4% and 16.7%, respectively.

## Introduction

The development and utilization of renewable resources have generated intensive interest due to the increasing demand for energy, the sharp decline in oil production and increasingly concerning environmental pollution, which is part caused by the burning of fossil fuels [1], [2]. Low-cost lignocellulosic biomass resources have been the subject of increased research interest in recent years as they can be used as raw materials for the production of ethanol-based fuels through microbial conversion [3], [4]. After hydrolysis, the lignocellulosic hydrolyzate contains a large amount of xylose, in addition to glucose [5], [6]. Glucose and the other six-carbon sugars can be converted to ethanol by yeast, such as *Saccharomyces cerevisiae*, as well as other traditional ethanol fermentation industrial yeast strains [7]; however, it has previously not be viable to use substantial quantities of xylose, a five-carbon sugar, in the fermentation process as the traditional yeast strains do not possess a metabolic pathway for xylose [8], [9], [10], [11]. Thus, a focus of research has been the production of ethanol fuel using engineered *Saccharomyces cerevisiae* strains, which can be achieved through microbial breeding. These strains are engineered to efficiently convert glucose and xylose to ethanol in a process that is adaptable to large-scale industrial production [12]. In addition, lignocellulosic hydrolyzate contains inhibitors that could inhibit microbial growth and reduce ethanol yield and productivity. Part of the focus of related research has shifted to inhibitor tolerance [13].For most wine and bottom-fermenting beer yeasts that are homothallic and have low sporulation ability, which require microaerophilic conditions for fermentation, breeding via hybridization can be achieved in practice by protoplast fusion [14], [15]. Previously, protoplast fusion has been used to breed wine and beer yeasts with high ester productivity and generate strains with improved abilities for lignocellulose degradation [13]. In this case the respiratory deficiency and nutritional requirements of the fusants were used as selective markers. However, such selective markers are not applied to industrial strains because they are prototrophic. Dominant selective markers are obviously useful for selecting hybrids when the protoplast fusion method is employed [16].

In this study, plasmids that contained drug resistance genes, G418 and blasticidin, and reporter genes, *adh*P–*GFP*–*adh*T, *adh*P–*gus*A–*adh*T were constructed for the selection of hybrids by protoplast fusion. Hybrids formed from transformants with each plasmid acquired resistance to both drugs and their colonies exhibited green fluorescence under fluorescence microscopy and a blue color when stained for β-glucuronidase (GUS) activity.

The effectiveness of protoplast fusion in combination with the drug resistance markers and reporter genes for selecting hybrids from wine yeasts without the need for auxotrophic or respiratory-defective markers was assessed. Using *Saccharomyces cerevisiae* W5, which is known to exhibit excellent fermentation performance, and *Candida shehatae* 20335, which is able to metabolically assimilate xylose under anaerobic conditions, as the parent strains, the technique was applied to the selection of engineered *Saccharomyces cerevisiae* strains that could efficiently utilize a mixed carbon source of xylose and glucose for the production of ethanol [17], [18], [19].

## Materials and Methods

### Yeast strains and growth conditions

*S. cerevisiae* W5, a diploid wild-type strain, was isolated from soil taken from Heilongjiang Province, China, and maintained in our laboratory [17]. Specific permission was not required during the research and sample collection, and the study did not involve endangered or protected species. *Escherichia coli* DH5*α* was purchased from Takara Biotechnology Co., Ltd. *Candida shehatae* ACCC 20335 was purchased from the Agricultural Culture Collection of China (ACCC). *Escherichia coli* DH5*α* were grown in lysogeny broth (LB) medium (1% tryptone, 0.5% yeast extract and 1% NaCl; *w*/*v*). *Saccharomyces cerevisiae* W5 and *Candida shehatae* 20335 were grown in yeast extract–peptone–dextrose (YEPD) liquid medium (2% peptone, 1% yeast extract and 2% dextrose; *w*/*v*) or in yeast extract–peptone–xylose (YEPX) liquid medium (2% peptone, 1% yeast extract and 2% xylose; *w*/*v*). In addition, strains were also grown in yeast extract–peptone–dextrose–sucrose (YEPDS) solid medium containing 2% agar and 17% sucrose (*w*/*v*).

Glucose fermentation medium (*w*/*v*; pH 5.0) consisted of 4% glucose, 0.5% yeast extract, 0.25% (NH_4_)_2_SO_4_, 0.25% KH_2_PO_4_, 0.025% MgSO_4_·7H_2_O and 0.025% CaCl_2_; xylose fermentation medium was the same as the glucose fermentation medium with 2% xylose instead of glucose; xylose and glucose co-fermentation medium (*w*/*v*; pH 5.0) consisted of 2.0% xylose, 4% glucose, 0.5% yeast extract, 0.25% KH_2_PO_4_, 0.25% (NH_4_)_2_SO_4_, 0.025% CaCl_2_ and 0.025% MgSO_4_·7H_2_O.

For the seed culture, one colony was inoculated into 20 mL/250 mL YEPD or LB liquid medium and incubated at 30°C or 37°C, respectively, for 12 h.

Yeasts are facultative anaerobic organisms, which require oxygen during the fermentation process. Three types of flask stopper (pinhole stopper, rubber stopper and aluminium foil) were trialed to control fermentation conditions in the flasks and ultimately the pinhole stopper was chosen according to the level of dissolved oxygen, ethanol yield and biomass production (data not shown). For the fermentation, seed cultures were inoculated into xylose, glucose or xylose–glucose co-fermentation medium at a concentration of 5% (*v*/*v*), using 125 mL working volume in 250 mL flasks with a pinhole stopper and fermented at 30°C with a shaking speed of 140 rpm for 100 h prior to the assessment of the ethanol yield and the use of carbon sources. All of the products were detected by HPLC.

### Construction of plasmids with drug resistance genes and reporter genes

**Figure 1:**
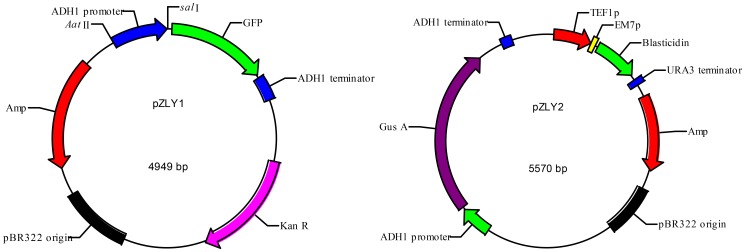
Structures of the pZLY1 plasmid (left) with the G418 resistance gene (*Kan*R) and *adh*1P–*GFP*–*adh*1t reporter gene, and the pZLY2 plasmid (right) with the blasticidin resistance gene (*bsd*) and *adh*1P–*gus*A–*adh*1t reporter gene. *adh*1P and *adh*1t represent, respectively, the promoter and terminator of the *adh*1 gene of *Saccharomyces cerevisiae*. *Kan*R represents the G418 resistance gene, *gus*A represents the *gus*A gene and *GFP* represent the *GFP* gene.

Plasmid pZLY1 was constructed by inserting a 500 bp *Aat*II–*Sal*I fragment containing the *adh*P promoter gene which was PCR amplified with the designed primers (ZLY-ADHp-1, 5′-GCG***GACGTC***CTATTGAAGTAATAATAGGCGCAT-3′ and ZLY-ADHp-2, 5′-GCG***GTCGAC***AGTTGATTGTATGCTTGGTATAG-3′). This amplicon contained *Aat*II and *Sal*I restriction sites from p406ADH1 (Addgene, # 15974) was cloned into the *Aat*II–*Sal*I gap of pKT0150 (Addgene, #8741) (Fig. 1), which contains a *GFP* reporter gene and G418 resistance gene.

Plasmid pZLY2 was constructed by inserting a 180 bp *Kpn*I–*Nde*I fragment containing the *adh*T terminator gene, which was PCR amplified with the following designed primers (ZLY-ADHt-1, 5′- GCG***GGTACC***GCGAATTTCTTATGATTTATGAT-3′ andZLY-ADHt-2, 5′- GCG***CATATG***GGTGTGGTCAATAAGAGCG-3′) that contained the *Kpn*I *and Nde*I restriction sites from pKT0150 into the *Kpn*I–*Nde*I gap of p406ADHI. Additionally, a 2000 bp *EcoR*I–*Xba*I *gus*A reporter gene which was digested from plasmid pBI121 (Biodee Biotechnology Corporation, Beijing, MP-091) by *EcoR*I and *Xba*I was inserted into the *ahd*P–*adh*T box and a 1000 bp *Aat*II fragment, with a blasticidin resistance gene (*bsd*). This was PCR amplified with the following primers: ZLY*-bsd-*1, 5′-GCG*G* ***ACGTC***CCCACACACCATAGCTTC-3′ and ZLY*-bsd-*2, 5′-GCG*G* ***ACGTC***GGGTAATAACTGATATAATTAAAT-3′, which contained *Aat*II restriction sites, from plasmid pYC6/CT (Invitrogen, V 825701), allowing pZLY2 to contain a *gus*A reporter gene and blasticidin resistance gene (Fig. 1).

The *gus*A gene (GI: 946149) is often used in plant transformation, as it encodes a hydrolytic enzyme β-glucuronidase that can catalyze the hydrolysis of x-gluc (5-bromo-4-chloro-3-indole glucuronide) (Solarbio Technology Corporation, Beijing, China, CAS 1141162-64-0) to a blue insoluble precipitate (indigo) that can be seen through microscopy or by the naked eyes; the coding region of the *gus*A gene is 1809 bp. The protocol for gus-activity staining was performed according to that described by Jefferson [20]: briefly, 10 mg of x-gluc was dissolved in 0.2 mL of 1, 2-dimethoxyethane and used as substrate mixture. A drop of the yeast suspension was put into 5 µL of substrate mixture and incubated at 37°C for 4 h to observe the appearance of blue precipitate.

### Transformation of pZLY1 and pZLY2 into *Candida shehatae* 20335 and *Saccharomyces cerevisiae* W5

Plasmids pZLY1 and pZLY2 were introduced into *Candida shehatae* 20335 and *Saccharomyces cerevisiae* W5, respectively, by a lithium acetate method [17]. A system without plasmid DNA was used as a negative control. Transformed *Candida shehatae* 20335 broth with pZLY1 (100 µL) and transformed *Saccharomyces cerevisiae* W5 broth with pZLY2 (50 µL) were spread on YEPX solid medium containing 600 µg mL^−1^ G418 (Calbiochem Corporation) and YEPD solid medium containing 40 µg mL^−1^ blasticidin (Invitrogen), respectively. The plates were then incubated at 30°C for 2–4 days.

A single colony growing on blasticidin resistance medium was inoculated into 20 mL/250 mL YEPD liquid medium and incubated overnight at 30°C with a shaking speed of 140 rpm. Expression of the *gus*A gene was detected upon appearance of a blue insoluble precipitate following the addition of x-gluc substrate, which demonstrated β-glucuronidase activity.

A single colony growing on the G418 resistance medium was inoculated into 20 mL/50 mL YEPX liquid medium and incubated overnight at 30°C with a shaking speed of 140 rpm. A drop of the yeast suspension was placed on a slide and the green fluorescence was observed under an inverted fluorescence microscope (Nikon, Eclipse TE 2000-S, Japan). The excitation and emission wavelengths for this detection were 488 nm and 507 nm.

### Protoplast fusion and the detection of fusants

The method described in the work by Ge et al. [17] was used to prepare the protoplasts of the transformants (pZLY2) for *Saccharomyces cerevisiae* W5 and the transformants (pZLY1) for *Candida shehatae* 20335. The two protoplast suspensions were mixed with a ratio of 1∶1 (*v*/*v*) at a concentration of approximately 1×10^7^ protoplasts mL^−1^ and centrifuged at 6000 rpm for 10 min at room temperature to harvest protoplasts. A polyethylene glycol (PEG) solution (1 mL; 30–40% PEG, *w*/*v*) was then carefully added to the protoplast suspension and kept warm for 2 h at 30°C. YEPDS liquid medium (5 mL) was added into the aforementioned mixed liquid and cultivated at 30°C with a shaking speed of 140 rpm for 2 h. The culture was then spread on the surface of YEPDS solid medium containing 600 µg mL^−1^ G418 and 40 µg mL^−1^ blasticidin.

A single colony growing on the resistance medium was inoculated into 20 mL/50 mL YEPX liquid medium and incubated overnight at 30°C with a shaking speed of 140 rpm to determine the expression of the *gus*A and *GFP* genes.

### Plasmid elimination in the fusants

The yeast episomal plasmid can be easily out-populated without selective pressure. Consequently, we removed the pZLY1 and pZLY2 plasmids after protoplast fusion to allow the fusants to ferment the carbon source without the need for selective pressure using the following method. Fusants were inoculated into 20 mL/50 mL YEPD liquid medium without G418 and blasticidin, incubated overnight at 30°C with a shaking speed of 140 rpm, subcultured the obtained yeast broth five times and planted on YEPD solid medium with and without G418 and blasticidin.

### Polymerase chain reaction (PCR) analysis and enzyme assays

Using the ZLYRHZ7 and *Saccharomyces cerevisiae* W5 genomic DNA extracted by TIANamp Yeast DNA Kit (Tiangen Biotech CO., Beijing, China, DP121221) as the template, amplification reactions for xylose metabolic enzymes, xylose reductase (XR, EC 1.1.1.21), xylitol dehydrogenase (XDH, EC 1.1.1.9) and xylulokinase (XKS, EC 2.7.1.17), were performed with a Mastercycler gradient cycler (Eppendorf GA, Germany) under the following conditions: a 10 µL aliquot of the reaction mixture was prepared with 0.2 µL *Taq* DNA polymerase (5 U mL^−1^), 0.8 µL dNTP mixture (2.5 mmol L^−1^), 0.8 µL MgCl_2_ (25 mmol L^−1^), 1 µL PCR buffer (10×, Mg^2+^ free), 1 µL each primer (1 pmol µL^−1^) and 0.2 µg DNA template. The sequences of the primers were as follows. YX-xyl1-1 and YX-xyl1-2: 5′-ACT***TCTAGA***TACATCCACAATGAGCCC-3′ and 5′-TTC***GGATC***TCTACGCAAAGAAAGCAG-3′, respectively, for *XYL1* (primers designed according to the *XYL1* sequence of *Candida shehatae*); ZMY-xyl2-1 and ZMY-xyl2-2: 5′-CCT***ACTAGT***ATGACTGCTAACCCTTCGCTC-3′ and 5′-CCG***ACTAGT***CTATTCAGGGCCATCAATGAAAC-3′, respectively, for *XYL2* (primers designed according to the *XYL2* sequence of *Candida shehatae*); and CXS-XKS1-1 and CXS-XKS1-2: 5′-CGG***ACTAGT***AGTACTTTAATGTTGTGTTCAGTAA-3′ and 5′-CGC***GTCGAC*** TTTAGATGAGAGTCTTTTCCAG-3′, respectively, for *XKS* (primers designed according to the *XKS* sequence of *Saccharomyces cerevisiae*). The PCR conditions were as follows: a preliminary step of 1 min at 94°C; 40 cycles of 1 min at 94°C, 1 min at 55°C, 1.5 min at 72°C and a final extension step of 10 min at 72°C. Enzymatic assays for the xylose metabolic enzymes (xylose reductase (XR, EC 1.1.1.21), xylitol dehydrogenase (XDH, EC 1.1.1.9) and xylulokinase (XKS, EC 2.7.1.17)) were performed in accordance with the published protocol [21].

### HPLC analysis

The production of ethanol, xylose, glucose and xylitol was determined by HPLC (Shimadzu LC-10ATvp) using a HPX-87H column (300 mm×7.8 mm, Aminex HPX-87H ion exclusion column) at 65°C with a refractive index detector (RID–10A). The eluent used was 0.005 M H_2_SO_4_ at a low flow rate of 0.8 mL min^−1^. The analysis time was 18 min. The injection volume of the sample was 20 µL. Commercially available ethanol (Tianjin Guangfu Technology Development Co., China), xylose (Shanghai Boao Biotechnology Co., China), glucose (Tianjin Kemiou Chemical Reagents Co., China) and xylitol (Institute of Guangfu Fine Chemical Industry of Tianjin) were used as standards.

## Results

### The construction of pZLY1 and pZLY2 and detection by enzymatic digestion

The pZLY1 and pZLY2 were successfully constructed and enzymatic digestion was used to ensure the reliability of the two plasmids. pZLY1 was digested with *Spe*I and *Sph*I, and two clear bands (2.8 kb and 2.1 kb) were obtained from the digestion. pZLY1 was digested with *EcoR*I and *Xba*I, and two clear bands (5.5 kb and 2167 bp, *gus*A) were again obtained from the digestion.

### Transformation of pZLY1 into *Candida shehatae* 20335 and fusion of protoplasts with dominant selective markers and reporter genes

**Figure 2:**
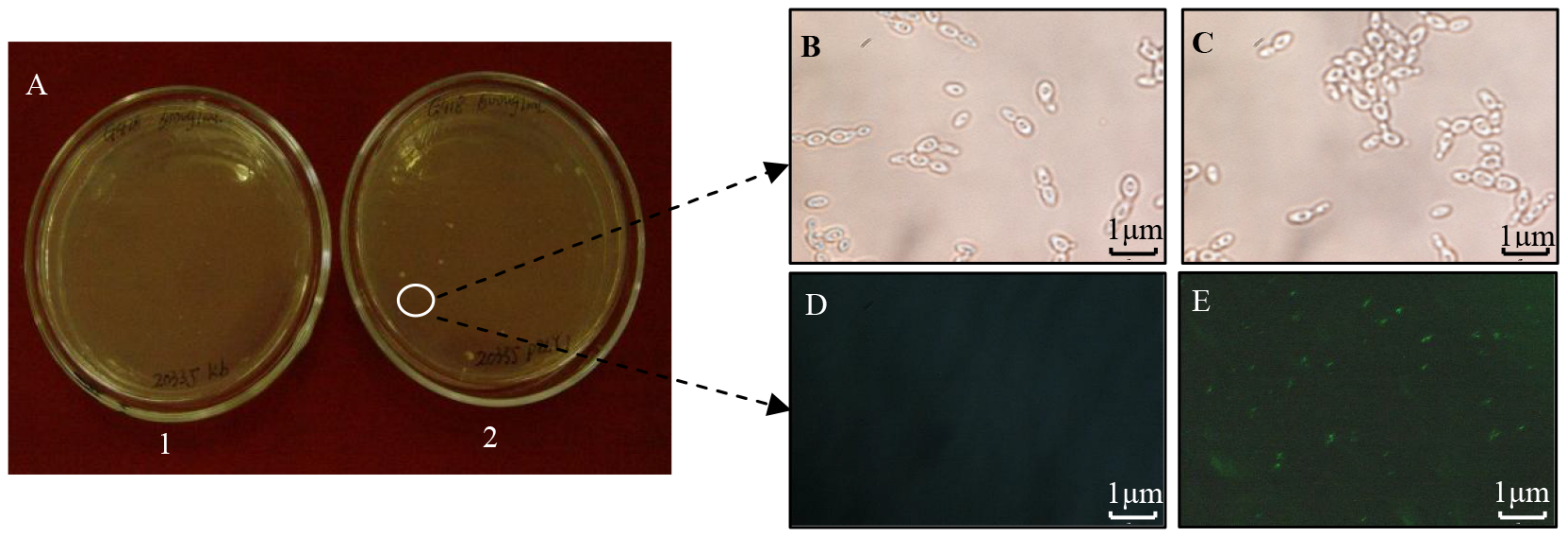
Transformation of pZLY1 into *Candida shehatae* 20335 and detection of the dominant selective marker (G418 resistance) and reporter genes (GFP gene). A. 1: Colonies were not observed on YEPX solid medium with G418 when *Candida shehatae* 20335 was not transformed by pZLY1; 2: several colonies grew on YEPX solid medium with G418 when *Candida shehatae* 20335 was transformed by pZLY1. B. *Candida shehatae* 20335 cells were observed under light microscopy (1600×). C. *Candida shehatae* 20335 transformants were observed under light microscopy (1600×). No morphological differences can be seen between the cells. D. *Candida shehatae* 20335 cells observed under fluorescence microscopy (1000×), showing GFP is not expressed in untransformed cells. E. *Candida shehatae* 20335 cells observed under fluorescence microscopy (1000×), showing GFP is expressed in transformants.

*Candida shehatae* 20335 transformed with pZLY1 yielded several colonies growing on the surface of the YEPX solid medium spread with 600 µg mL^−1^ G418 (Fig. 2). In contrast, no untransformed *Candida shehatae* 20335 colonies grew on the surface of YEPX solid medium spread with 600 µg mL^−1^ G418. One colony was selected and incubated in YEPX liquid medium at 30°C with a shaking speed of 140 rpm. Cells were assessed for their green fluorescence by microscopy (Nikon, Eclipse TE 2000–S, Japan).

### The screening and identification of *Saccharomyces cerevisiae* W5 transformants

**Figure 3:**
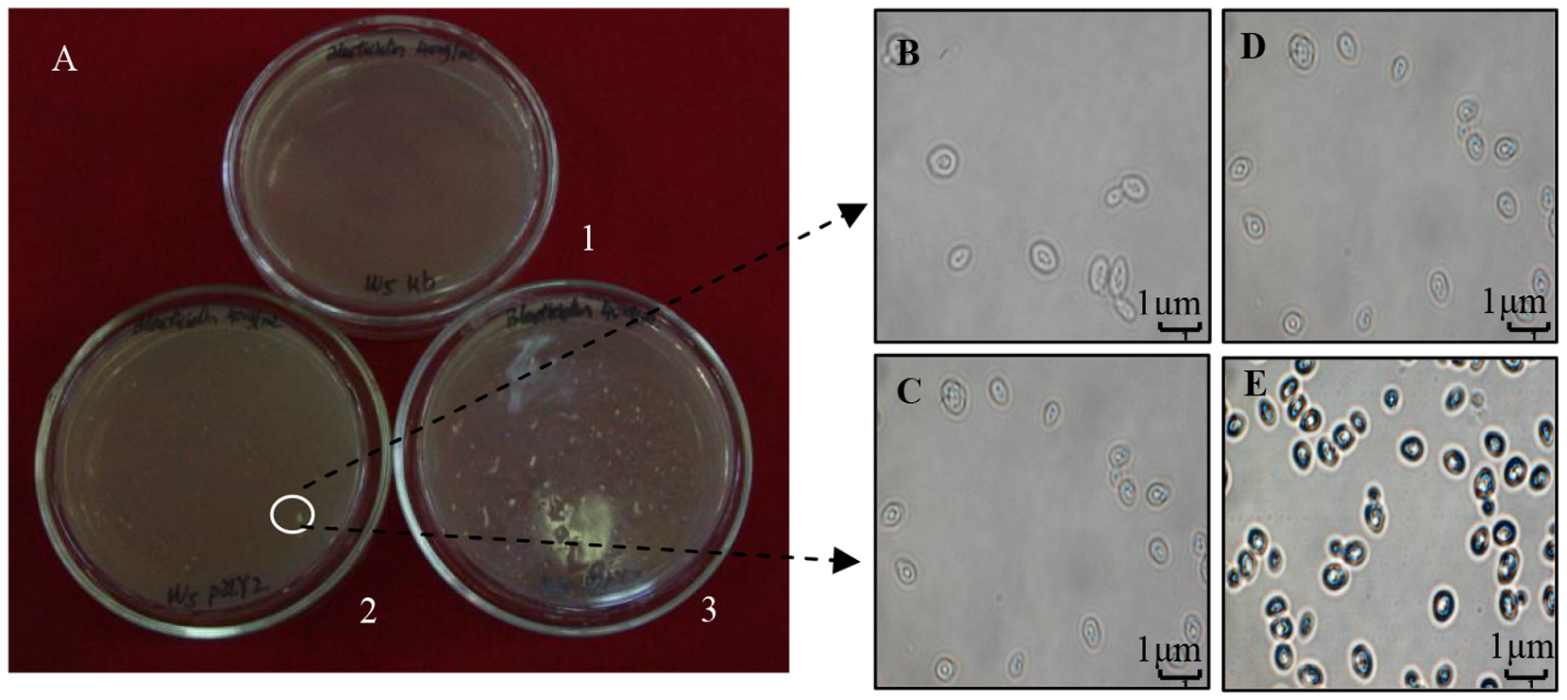
Transformation of pZLY2 into *Saccharomyces cerevisiae* W5 and detection of the dominant selective marker (blasticidin resistance) and reporter gene (*gus* gene). A. 1: No colonies grow on YEPD solid medium with blasticidin when *Saccharomyces cerevisiae* W5 was not transformed by pZLY2; 2 and 3: several colonies grow on YEPD solid medium with blasticidin when *Saccharomyces cerevisiae* W5 was transformed by pZLY2. B. *Saccharomyces cerevisiae* W5 cells observed under light microscopy (1600×). C GUS-stained *Saccharomyces cerevisiae* W5 cells, as observed by light microscopy (1600×). D. Positive transformants of *Saccharomyces cerevisiae* W5 observed by light microscopy (1600×). No morphological differences can be seen between the cells. E. GUS-stained transformants of *Saccharomyces cerevisiae* W5, as observed by light microscopy (1600×), showing the blue cells.

*Saccharomyces cerevisiae* W5 transformed with pZLY2 yielded several colonies growing on the surface of YEPD solid medium spread with 40 µg mL^−1^ blasticidin (Fig. 3). In contrast, untransformed *Saccharomyces cerevisiae* W5 colonies did not grow on the surface of YEPD solid medium with 40 µg mL^−1^ blasticidin. One colony was selected and incubated in YEPD liquid medium containing 40 µg mL^−1^ blasticidin at 30°C with a shaking speed of 140 rpm. Resulting cells were blue when stained for β- glucuronidase activity (encoded by the *gus*A gene), which was observed by microscopy (Nikon, Eclipse TE 2000–S, Japan). Negative control cells were not blue.

**Figure 4:**
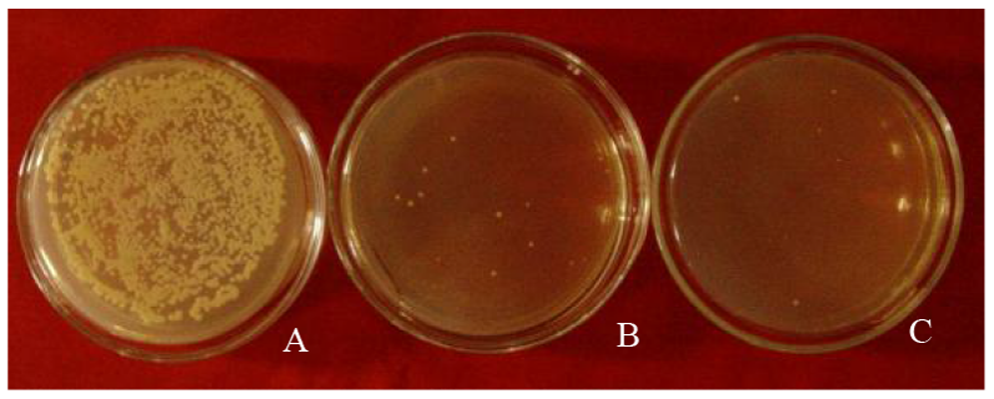
Screening of protoplast fusants. A. Many colonies are found to grow on YEPDS regenerated solid medium when protoplast fusants are spread on it. B and C. Several colonies grow on YEPDS regenerated solid medium with blasticidin and G418.

These results indicate that drug resistance genes and reporter genes in both plasmids function normally. Protoplasts from the resulting transformants were fused as described in the Material and Methods section, and several colonies were found on the selection medium (Fig. 4).

### Evaluation of fusion products

To confirm whether the fusion products retain both plasmids, they were grown on a selection medium and examined for the expression of reporter genes by plate analyses and fluorescence microscopy. All colonies grew on the medium containing G418 and blasticidin and exhibited green fluorescence, as observed by fluorescence microscopy, or a blue color when stained for β-glucuronidase activity (encoded by the *gus*A gene), which indicated that the cells contained both plasmids pZLY1 and pZLY2.

### Ethanol production by fusants with different feed sources

After protoplast fusion by *Candida shehatae* 20335 and *Saccharomyces cerevisiae* W5 transformants, ten fusants were obtained and one fusant, ZLYRHZ7, was selected for further analysis. ZLYRHZ7 was cultured in YEPD or YEPX liquid medium and inoculated into xylose fermentation medium, glucose fermentation medium or xylose–glucose co-fermentation medium.

The resulting supernatants were collected every 12 h during 100 h fermentation for analysis of the ethanol production by HPLC. Untransformed yeast strains *Candida shehatae* 20335 and *Saccharomyces cerevisiae* W5 were used as controls. All data were analyzed by SPSS one-dimensional Duncan analysis of variance.

ZLYRHZ7, *Candida shehatae* 20335 and *Saccharomyces cerevisiae* W5 produced 9.31, 7.98 and 0 g L^−1^ ethanol, respectively, when xylose was used as the sole carbon source, which indicated that ZLYRHZ7 can utilize xylose for the production of ethanol (Table 1). Furthermore, ZLYRHZ7, *Candida shehatae* 20335 and *Saccharomyces cerevisiae* W5 produce 17.04, 15.40 and 17.23 g L^−1^ ethanol, respectively, when glucose was used as the sole carbon source, indicating that ZLYRHZ7 has the same glucose fermentation ability as *Saccharomyces cerevisiae* W5 (Table 2).

**Table 1:** Fermentation of xylose as the sole carbon source by *Candida shehatae* 20335, *Saccharomyces cerevisiae* W5 and ZLYRHZ7 transformants.

Strains	Xylose content (g L^−1^)	Residual xylose (g L^−1^)	Xylose utilization (g L^−1^)	Xylitol production (g L^−1^)	Ethanol production (g L^−1^)	Ethanol yield (g g^−1^)
W5	25.25±0.01	22.13±0.151	3.12±0.15	0±0.00	0±0.00c	0±0.000c
20335	25.25±0.01	0±0.00	25.25±0.00	0±0.00	7.98±0.15b	0.316±0.003b
ZLYRHZ7	25.25±0.01	0±0.00	25.25±0.00	0±0.00	9.31±0.17a	0.369±0.004a

Data are expressed as the mean values ± standard deviation of at least three independent experiments. The different letters in the same column of the data indicated in the p<0.05 level of significant difference.

**Table 2:** Fermentation of glucose as the sole carbon source by *Candida shehatae* 20335, *Saccharomyces cerevisiae* W5 and ZLYRHZ7 transformants.

Strains	Glucose content (g L^−1^)	Residual glucose (g L^−1^)	Glucose utilization (g L^−1^)	Xylitol production (g L^−1^)	Ethanol production (g L^−1^)	Ethanol yield (g g^−1^)
W5	41.47±0.01	0±0.001	41.47±0.01	0±0.00	17.23±0.11a	0.415±0.00a
20335	41.47±0.01	0±0.001	41.47±0.01	0±0.00	15.40±0.11b	0.371±0.00c
ZLYRHZ7	41.47±0.01	0±0.001	41.47±0.01	0±0.00	17.04±0.19a	0.411±0.00b

Data are expressed as the mean values ± standard deviation of at least three independent experiments. The different letters in the same column of the data indicated in the p<0.05 level of significant difference.

**Figure 5:**
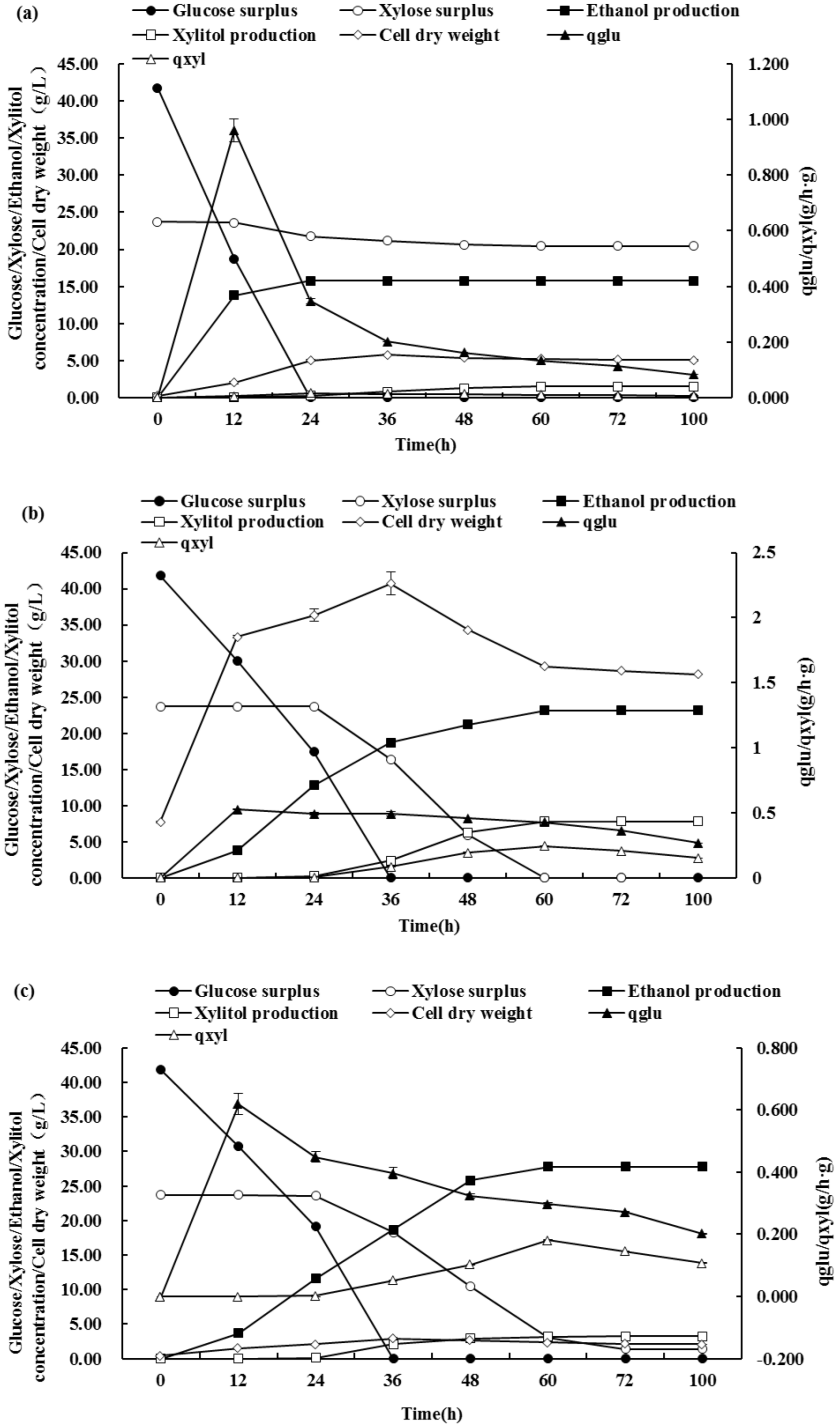
Changes in the concentrations of residual glucose (black circle), residual xylose (open circle), ethanol production (black square), xylitol production (open square), cell dry weight (open rhombus), qglu (black triangle) and qxyl (open triangle) in the presence of *Saccharomyces cerevisiae* W5 (a), *Candida shehatae* 20335 (b) and ZLYRHZ7 (c) during fermentation in the presence of both glucose and xylose as carbon sources. Data are expressed as the mean ± standard deviation of three independent experiments.

Fig. 5 illustrates the fermentation kinetics of ZLYRHZ7, *Candida shehatae* 20335 and *Saccharomyces cerevisiae* W5, including ethanol and xylitol production and glucose and xylose consumption. ZLYRHZ7 produced higher levels of ethanol compared with *Saccharomyces cerevisiae* W5, whereas the ethanol production between ZLYRHZ7 and *Candida shehatae* 20335 was not obvious.

*Saccharomyces cerevisiae* W5 consumed available glucose by 24 h that corresponded to its highest ethanol output (15.74 g L^−1^), while, both *Candida shehatae* 20335 and fusant ZLYRHZ7 could consumed available glucose by 36 h, which was 12 h slower than *Saccharomyces cerevisiae* W5. At later time points, *Candida shehatae* 20335 and fusant ZLYRHZ7 continued to use xylose and produced ethanol and xylitol, and reached their highest ethanol output (23.15 g L^−1^ for 20335 and 27.77 g L^−1^ for ZLYRHZ7) at 54 h and 60 h, respectively.

After a 100 h fermentation period, both *Candida shehatae* 20335 and fusant ZLYRHZ7 showed the same trend in the utilization of xylose and glucose. They both consumed glucose completely by 36 h and used more xylose than *Saccharomyces cerevisiae* W5. But *Candida shehatae* 20335 used up xylose by 54 h while fusant ZLYRHZ7 could only consumed 22.34 g L^−1^ xylose by the end of the experiment.

Table 3 compares the fermentation behavior of ZLYRHZ7, *Candida shehatae* 20335 and *Saccharomyces cerevisiae* W5 when the fermentation time was set at approximately 65 h, including the ethanol and xylitol production and glucose and xylose consumption. Clear differences can be seen between these three strains, *i.e.*, ZLYRHZ7 produced significantly higher levels of ethanol (27.77 g L^−1^) compared with *Saccharomyces cerevisiae* W5 (15.74 g L^−1^) (*p*<0.05). While the ethanol output of ZLYRHZ7 was not comparable to *Candida shehatae* 20335, the ethanol yield differed, with *Candida shehatae* 20335 producing a yield of 0.353 g g^−1^ and ZLYRHZ7 producing a yield of 0.424 g g^−1^. The reason for this difference is that, when using *Candida shehatae* 20335, some of the carbon source is used to produce xylitol (output 7.82 g L^−1^).

**Table 3:** Fermentation of glucose–xylose by *Candida shehatae* 20335, *Saccharomyces cerevisiae* W5 and ZLYRHZ7 transformants.

Strains	Glucose content (g L^−1^)	Xylose content (g L^−1^)	Glucose utilization (g L^−1^)	Xylose utilization (g L^−1^)	Xylitol production (g L^−1^)	Ethanol production (g L^−1^)	Ethanol yield (g g^−1^)
W5	41.79±0.01	23.70±0.01	41.79±0.01	3.23±0.12	1.49±0.11b	15.74±0.170c	0.240±0.001c
20335	41.79±0.01	23.70±0.01	41.79±0.01	23.70±0.01	7.82±0.10a	23.15±0.210b	0.353±0.0022b
ZLYRHZ7	41.79±0.01	23.70±0.01	41.79±0.01	22.34±0.19	3.20±0.14ab	27.77±0.200a	0.424±0.002a

Data are expressed as the mean values ± standard deviation of at least three independent experiments. The different letters in the same column of the data indicate the level of significant differences at p<0.05.

However, *Saccharomyces cerevisiae* W5 can also assimilate a small proportion of xylose and it subsequently produced a low xylitol yield (1.49 g L^−1^). ZLYRHZ7, similar to *Candida shehatae* 20335, is able to assimilate xylose and the xylose utilization and xylitol production were 22.34 and 3.20 g L^−1^, respectively, which is significantly higher than *Saccharomyces cerevisiae* W5 (3.23, 1.49 g L^−1^) (*p*<0.05). While the xylitol production of ZLYRHZ7 was 59% lower than that of *Candida shehatae* 20335, the metabolism pathway in ZLYRHZ7 is consequently more unimpeded. The ethanol yield of ZLYRHZ7 was 0.424 g g^−1^, *i.e.*, 43.4% more than that generated by *Saccharomyces cerevisiae* W5 (0.240 g g^−1^) and 16.7% more than that generated by 20335 (0.353 g g^−1^). However, ZLYRHZ7 was unable to completely ferment xylose, with 5.7% unused xylose remaining after 100 h and a corresponding ethanol production of 27.77 g L^−1^.

### PCR of *XYL1*, *XYL2* and *XKS*

**Figure 6:**
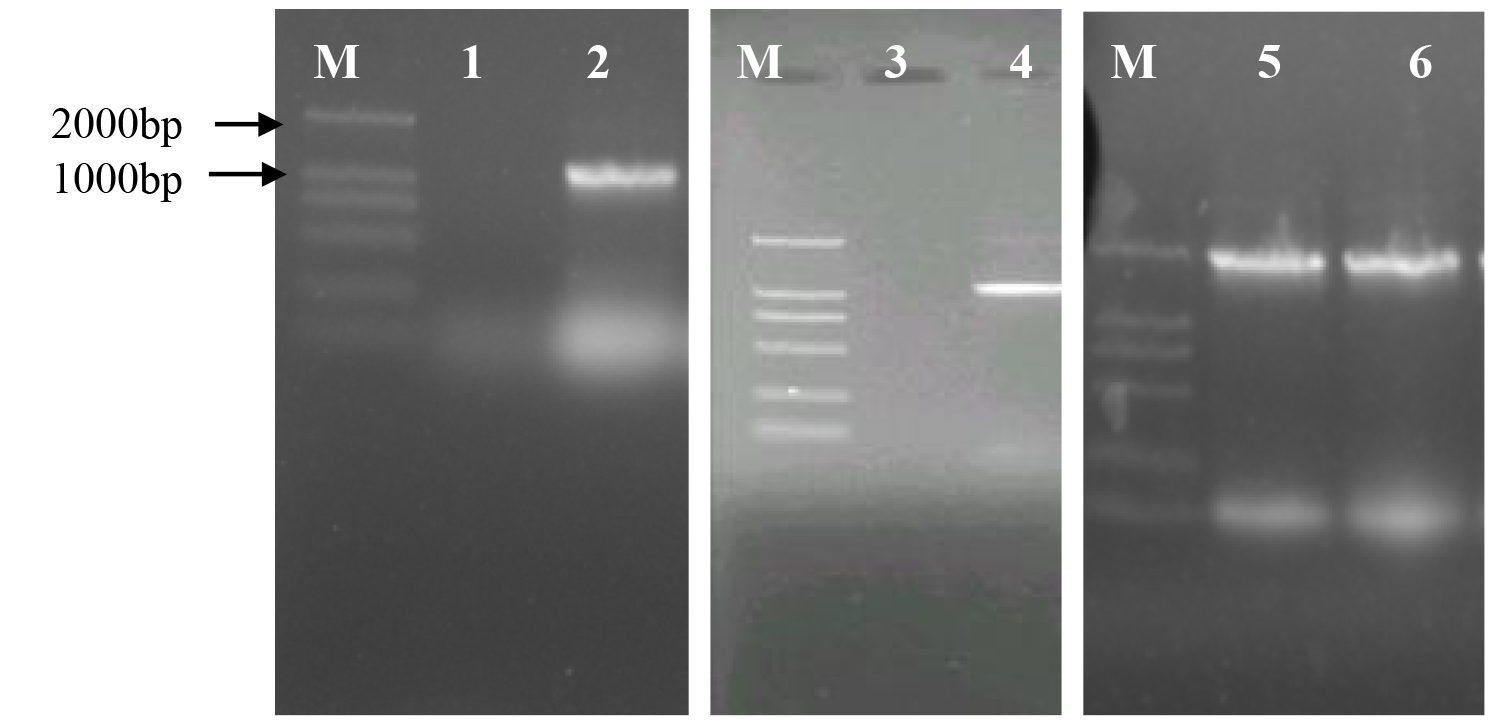
The amplified genes of xyl1, xyl2 and XKS using the chromosomes of ZLYRHZ7 and *S. cerevisiae* W5 as the template. M is the marker; lanes 1, 3 and 5 are the amplified genes of xyl1, xyl2 and XKS of *S. cerevisiae* W5; lanes 2, 4 and 6 are the amplified genes of xyl1, xyl2 and XKS of ZLYRHZ7.

The *XYL1*, *XYL2* and *XKS* were amplified using the genomic DNA of ZLYRHZ7 as the template. The results showed that the three genes were amplified and indicated that the protoplast fusion was successful (Fig. 6). However, when using the *Saccharomyces cerevisiae* W5 genomic DNA as the template, no amplification of *XYL1*, *XYL2* occurred. *S. cerevisiae XKS* could be amplified in both ZLYRHZ7 and *Saccharomyces cerevisiae* W5, which indicated that fusant ZLYRHZ7 combined the genomes of *Saccharomyces cerevisiae* W5 and *Candida shehatae* 20335.

### Enzyme activities

The protoplast fusion and xylose-fermenting yeast strain ZLYRHZ7 was generated in this study. The XR, XDH and XKS activities of different strains were measured during their fermentation (48 h) (Table 4). The XR, XDH and XKS activities of ZLYRHZ7 were found to be increased by more than 180%, 25% and 81% compared to *Saccharomyces cerevisiae* W5, and by approximately 34%, 42% and 64% compared to *Candida shehatae* 20335, indicating that the three ZLYRHZ7 genes are effectively expressed.

**Table 4:** XR, XDH and XKS activities in different cell extracts (48 h).

Strains	Enzyme activities (U mg^−1^)		
	XR	XDH	XKS
W5	2.170±0.057	15.71±0.01	75.36±0.00
20335	4.527±0.010	13.83±0.01	83.32±0.01
ZLYRHZ7	6.070±0.020	19.57±0.48	136.52±0.03

## Discussion

Although traditional breeding methods have succeeded in generating many industrial, ethanol-producing yeast strains, they are time-consuming and high-cost processes. However, protoplast fusion is a technique that allows for the recombination of several genomes simultaneously at different sites without the necessity for detailed genomic information [22], [23].

We previously attempted the use of genome shuffling technology to introduce *XYL1*, *XYL2* and *XKS* into a single *Saccharomyces cerevisiae* chromosome and a high ethanol-producing *Saccharomyces cerevisiae* GS3-10 fusant was obtained, which produced 26.65 g L^−1^ ethanol, which was 47.1% higher than the ethanol production of *Saccharomyces cerevisiae* W5 [17]. However, this method does have some drawbacks as it can be time-consuming and requires laborious screening.

To address this need for a facile breeding method, in this study we constructed two episomal plasmids, pZLY1 and pZLY2, which could be easily removed. While plasmid pZLY1 contains a G418 resistance marker and *GFP* reporter gene, plasmid pZLY2 contains a blasticidin resistance marker and *gus*A reporter gene. We introduced the pZLY1 and pZLY2 plasmids into *Candida shehatae* 20335 and *Saccharomyces cerevisiae* W5, respectively, and obtained the corresponding transformants by a lithium acetate transformation method. Protoplast fusants were then obtained using the four markers through a protoplast fusion method.

There are a number of advantages of using this method, when compared to other protoplast fusion screening methods: (1) since the plasmids were eliminated, we achieved optimal fermentation abilities and advantageous characteristics of the fusants; in addition, this ensured the nutritional requirements of the fusants were met without the existence of a resistance drug. (2) As the G418 and blasticidin resistance markers are commonly used in *Saccharomyces cerevisiae* and the expression of *GFP* and *gus*A reporter genes can be easily detected by fluorescence microscopy and histochemical detection, there is little need for expensive equipments and the cost of this method could be easily met by industry should the method be scaled up.

Importantly, the characteristics of the fusants obtained in this study are much improved compared to those obtained through the inactivated protoplast fusion method. In this study, the parental genomic DNA of ZLYRHZ7 constructed a relatively unimpeded xylose metabolism pathway, attributed to the lower amount of xylitol found and showed a better fermentation performance. The fusant was found to have the highest ethanol yield (0.424 g g^−1^) using a mixed carbon source.

In our previous work we introduced a xylose metabolic pathway into *Saccharomyces cerevisiae* W5 using genome shuffling technology. Using this previous approach, we achieve an ethanol yield of 0.40 g g^−1^ for the GS3-10 fusant. In this work we achieved a comparable, high ethanol yield using a much simpler and cost effective method. Nakazawa et al. [24] have also previously used this method with wine yeasts that had no genetic markers and six hybrid strains were achieved.

The fusant achieve in this study (ZLYRHZ7) has the genetic characteristics of the parental strains, *Saccharomyces cerevisiae* W5 and *Candida shehatae* 20335, and also initially carries pZLY1 and pZLY2 plasmids. In order to eliminate the influence of the plasmids on ZLYRHZ7, we inoculated the fusants into YEPD liquid medium without G418 and blasticidin and passaged five times. The episomal plasmids are easily lost in the absence of selective pressure conditions and, although the passaged strains can grow on medium without G418 and blasticidin, they cannot grow on medium in the presence of G418 and blasticidin. Ten of the twelve fusants were unable to grow on medium in the presence of G418 and blasticidin (data not shown). However, after performing our fermentation study, we found that these ten strains were still able to produce ethanol and ZLYRHZ7 showed stable characteristics, with ethanol production levels of 27.78, 27.96, 27, 15, 27.55 and 27.38 g L^−1^.

Theoretically, the genome of ZLYRHZ7 combined the genomes of *Saccharomyces cerevisiae* W5 and *Candida shehatae* 20335; this was confirmed from the fermentation results. Firstly, the xylitol production of ZLYRHZ7 and *Candida shehatae* 20335 with mixed carbon sources were 3.20 and 7.82 g L^−1^, respectively, which indicated that XR was in ZLYRHZ7. This enzyme had to come from *Candida shehatae* 20335, as there was no XR in *Saccharomyces cerevisiae* W5. Then, the ethanol production of ZLYRHZ7 and *Candida shehatae* 20335 with mixed carbon sources were 27.77 and 23.15 g L^−1^, respectively, indicating that the two strains both had XDH, and this enzyme could convert xylitol into xylulose and ethanol (there was no XDH in *Saccharomyces cerevisiae* W5). The XR and XDH activities in ZLYRHZ7 were higher than in *Candida shehatae* 20335 (Table 4), and the xylitol produced in ZLYRHZ7 was converted into xylulose, so the xylitol production in ZLYRHZ7 was lower than in *Candida shehatae* 20335. Above all, the genomic content of each parent contributes to the fermentation performance of ZLYRHZ7.

## Conclusions

This study constructed two plasmid vectors with resistance markers and reporter genes selection markers and selected protoplast fusants that used *Saccharomyces cerevisiae* W5 and *Candida shehatae* 20335 as parent strains. The fusants could assimilate xylose and glucose as the carbon source for ethanol fermentation and the ethanol yield was assessed by HPLC. The fusant ZLYRHZ7 had an ethanol yield using the mixed carbon source of 0.424 g g^−1^, which was better than the original strains with 0.240 g g^−1^ (W5) and 0.353 g g^−1^ (20335). This indicates an improvement in the ethanol yield of 43.4% (W5) and 16.7% (20335), respectively.
